# Association between childhood socioeconomic position and sports group participation among Japanese older adults: A cross-sectional study from the JAGES 2010 survey

**DOI:** 10.1016/j.pmedr.2020.101065

**Published:** 2020-02-17

**Authors:** Mitsuya Yamakita, Satoru Kanamori, Naoki Kondo, Toyo Ashida, Takeo Fujiwara, Taishi Tsuji, Katsunori Kondo

**Affiliations:** a College of Liberal Arts and Sciences, Kitasato University, Japan; b Department of Preventive Medicine and Public Health, Tokyo Medical University, Japan; c Human Resource Management Department, ITOCHU Techno-Solutions Corporation, Japan; d Departments of Health and Social Behavior/Health Education and Health Sociology, School of Public Health, University of Tokyo, Japan; e Institute of Social Science, University of Tokyo, Japan; f Department of Global Health Promotion, Tokyo Medical and Dental University, Japan; g Center for Preventive Medical Sciences, Chiba University, Japan; h Center for Gerontology and Social Science, National Center for Geriatrics and Gerontology, Japan

**Keywords:** Childhood disadvantage, Sports participation, Education, Older people, Life course

## Abstract

Sports group participation may have greater effects on health outcomes than exercising alone. Unhealthy lifestyles were reported to be specifically associated with lower socioeconomic positions (SEPs), and child poverty and the bipolarization of sports participation are currently major policy concerns in children. However, it remains unclear whether childhood SEP has any long-latency effect on sports group participation among older Japanese. Data were obtained from the Japan Gerontological Evaluation Study 2010 project, which used self-report questionnaires to survey individuals aged ≥65 years without disability from 27 municipalities (n = 23,320). According to their answers, respondents were assigned to one of three SEP groups: high, middle, or low. Poisson regression with robust variance and multiple imputations was used to examine the association between childhood SEP and sports group participation. After adjusting for health-related factors, low childhood SEP was negatively associated with sports group participation in men (prevalence ratio [PR] = 0.82, 95% confidence interval [CI] = 0.74–0.91) and women (PR = 0.88, 95% CI = 0.80–0.97). The PR was greatly attenuated after adjusting for educational attainment in both men (PR = 0.92, 95% CI = 0.83–1.02) and women (PR = 0.98, 95% CI = 0.89–1.08), and the significant association disappeared. Low childhood SEP is thus associated with lower sports group participation among older Japanese, though this may be attenuated by education. These findings suggest that it may be necessary to consider childhood SEP and the importance of education to increase sports group participation at an older age.

## Highlights

• Low childhood socioeconomic position was linked to lower sports participation in Japanese older adults.
• This association was attenuated by education.
• Education had a stronger association than adult socioeconomic position.
• Reducing child poverty and enriching education may increase sports group participation.

## 1 Introduction

Childhood socioeconomic position (SEP) are powerful predictors of health outcomes such as cardiovascular mortality, all-cause mortality (Cohen et al., 2010), and others (Galobardes et al., 2008, Rocha et al., 2019, Tamayo et al., 2010). Thus, since socioeconomic inequalities in childhood health have multiple adverse health consequences in later life, tackling these inequalities is an important public policy goal. Unhealthy lifestyles are reported to be correlated to a lower SEP (Foster et al., 2018). Several systematic reviews suggest that childhood SEP is an important determinant of later physical activity (PA) in adulthood (Elhakeem et al., 2015, Juneau et al., 2015). However, as there are fewer studies on older adults, it is not still completely understood whether childhood SEP contributes to later PA in old age.

The health benefits of PA for people of all ages are widely established (Lee et al., 2012, World Health Organization, 2018). In particular, sports group participation includes not only physiological benefits through increased PA but also psychological and social benefits through social participation beyond improvements attributed to individual types of PA (Eime et al., 2013, Farrance et al., 2016). A large cohort study of Japanese older adults showed that people who participated in sports groups showed a greater likelihood of avoiding functional disability than those who exercised alone (Kanamori et al., 2012), and that increasing the frequency of sports group participation alleviates worsening depressive symptoms compared with increasing daily walking time (Tsuji et al., 2017). In addition, another longitudinal study revealed that exercising with others has a more positive impact on mental well-being than exercising alone (Harada et al., 2019). Moreover, randomized controlled trials showed that social relations in exercise programs improved loneliness in older adults (Ehlers et al., 2017). This growing evidence suggests the possibility that sports group participation has a greater effect on health than PA alone, such as walking.

Understanding the association of childhood SEP with sports group participation among older adults may provide important insights into the pathways through which socioeconomic inequalities lead to life-long adverse health consequences. It is particularly meaningful to examine this association among the Japanese people with a high relative child poverty rate (one in seven) (Ministry of Health, Labour and Welfare, 2018, OECD, 2019), along with the ongoing bipolarization of sports participation (Sasakawa Sports Foundation, 2015). However, most previous studies were based on American or European populations and have shown the association with PA, and no studies have examined whether childhood SEP later contributes to sports group participation among older Japanese adults. Therefore, this study aimed to examine the association between childhood SEP and sports group participation among Japanese older adults.

## 2 Methods

### 2.1 Study participants

**Fig. 1:**
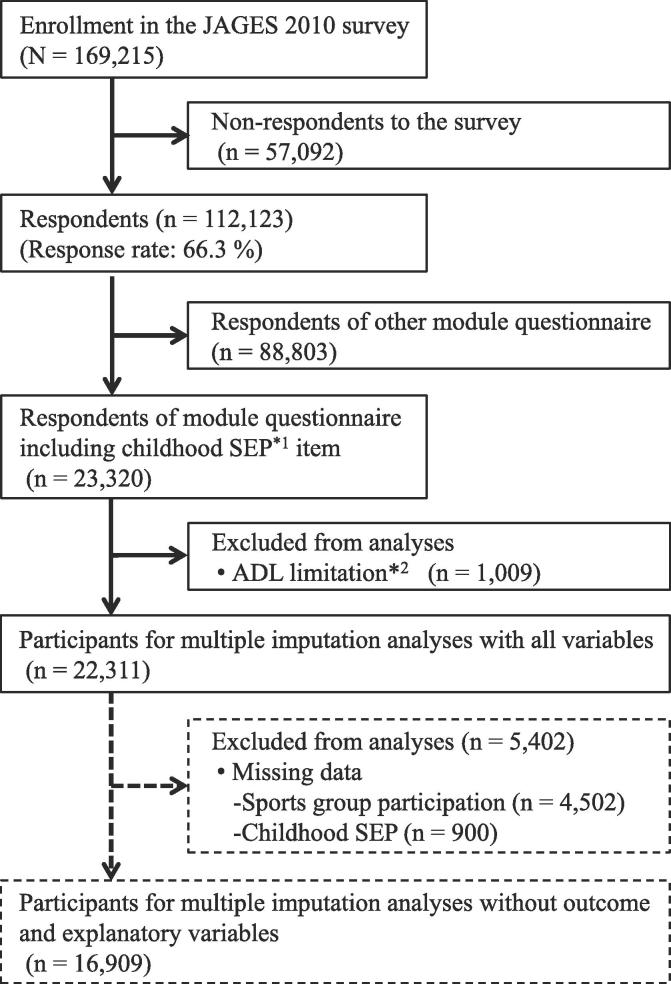
Flow chart of participant selection in the Japan Gerontological Evaluation Study (JAGES) 2010 survey. *1 SEP: socioeconomic position. *2 ADL: activities of daily living.

This study utilized data from the Japan Gerontological Evaluation Study (JAGES) (Kondo and Rosenberg, 2018). The JAGES was established in 2010 to evaluate the social determinants of healthy aging among non-disabled people aged 65 or above, sampled from 31 municipalities in 12 of the 47 prefectures throughout Japan. From August 2010 to January 2012, a self-administered questionnaire was mailed to 169,215 community-dwelling individuals aged 65 or above who were physically and cognitively independent and living independently. Random sampling was used in the 16 large municipalities, while the questionnaire was sent to all eligible residents in the 15 small municipalities. Of the eligible participants, 112,123 returned the questionnaire (66.3% response rate) (Fig. 1). The JAGES questionnaire consisted of basic questions to be answered by all respondents, as well as five separate modules that were randomly allocated to participants (20% probability for each module). Of these, one data module, which included items related to childhood SEP (23,320 respondents; 10,657 men and 12,663 women), was used in this study. In this cross-sectional study, the analysis included 22,311 participants (10,276 men and 12,035 women) after excluding participants who reported limitations in activities of daily living (n = 1,009), defined as being unable to walk, take a bath, or use the toilet without assistance, and who were mistakenly included in the study (Fig. 1). The JAGES protocol was reviewed and approved by the Ethics Committee on Research of Human Subjects at Nihon Fukushi University (Approval No. 10–05). Written informed consent was assumed from the voluntary return of the questionnaire.

### 2.2 Participation in sports groups

This was assessed using the following question: “How often do you participate in a sports group or club?” Those who answered, “almost every day,” “2 or 3 times a week,” “once a week,” “once or twice a month,” “a few times a year,” and “never.” To examine the differences between people who have never been interested in sports group participation and those who have participated in sports groups, the participants were classified into two groups: “Non-participants (never)” and “Participants (other than never),” in accordance with a previous study (Ashida et al., 2016). Sports group participation included participation in not only team sports, but also sports organizations.

### 2.3 Childhood SEP

This was retrospectively assessed by recalled subjective SEP using the following question: “How would you rate your socioeconomic status at the age of 15 years according to standards at that time?” Responses were arranged on a five-point Likert scale: “high,” “middle-high,” “middle,” “middle-low,” and “low.” These responses were allocated to three categories: high (including “high” and “middle-high”), middle, and low (including “middle-low” and “low”) to maximize the sample size for each category. This method has previously been validated using siblings’ data (Ward, 2011). Moreover, recalled subjective SEP in childhood has shown a good correlation with adult height (as a proxy for childhood nutrition) and/or homeownership (Fujiwara et al., 2014, Tani et al., 2016, Yanagi et al., 2017).

### 2.4 Covariates

Based on previous studies (Kanamori et al., 2012, Tani et al., 2016, Yamakita et al., 2015, Yanagi et al., 2017), the following variables were used as covariates. As health-related factors, age (continuous variables), current medical treatment (yes/no), instrumental activities of daily living (IADL), self-rated health, depression, body mass index (BMI), smoking status (non-smoker, ex-smoker, current smoker), alcohol intake (non-drinker, ex-drinker, or current drinker), marital status (married, widowed, divorced, single), providing emotional social support (yes/no), and receiving emotional social support (yes/no) were included. IADL was assessed using the Tokyo Metropolitan Institute of Gerontology Index of Competence (“good” [5 points] or “poor” [0–4 points]) (Koyano et al., 1991). Self-rated health is a subjective indicator that reflects the overall health status. In this study, we evaluated this indicator by asking the following question: “How is your current health status?” The possible responses were “excellent,” “good,” “fair,” and “poor.” Depression was measured using the short version of the Geriatric Depression Scale–15 (Japanese version) and was categorized into three groups (“no” [0–4 points], “mild” [5–9 points], “moderate to severe” [10–15 points]) (Nyunt et al., 2009, Schreiner et al., 2003). Body mass index (BMI) was calculated from self-reported height and weight (kg/m^2^).

### 2.5 Mediators

According to a previous study, height (Fujiwara et al., 2014), educational attainment (Frenz et al., 2017, Galobardes et al., 2008, Lawlor et al., 2006), and adulthood SEP (Cheval et al., 2018, Umeda et al., 2015) were used as potential mediators. These variables were assessed from the self-reported questionnaire. Height was used as a proxy for the childhood nutritional environment and disease history (Silventoinen, 2003), and was categorized into five groups at 5 cm intervals for each sex, as shown in Table 1, Table 2, Table 3. Previous studies confirmed a high correlation between self-reported and measured height among older people in Australia (Ng et al., 2011). Educational attainment was assessed categorized into three groups by years of schooling (<10, 10–12, ≥13 years). As indicators of adulthood SEP, current annual household income, which reflects SEP in old-age, and longest-held occupation, which reflects SEP in middle-age, were included. Annual household income was calculated by dividing household income by the square root of the number of household members and categorized into three groups (<2.00, 2.00–3.99, ≥4.00 million yen). Since previous studies have confirmed that the Japanese managerial/professional class appears to potentially experience a higher CHD risk compared to other occupations (Zaitsu et al., 2019), longest-held occupations were categorized into three groups: non-manual occupations (professional, technical, managerial work), manual occupations (clerical, sales/service, skilled/labor or agriculture/forestry/fishery worker, other), and no occupation (Tani et al., 2016).

**Table 1:** Characteristics of participants of the Japan Gerontological Evaluation Study (JAGES) 2010 survey (n = 22,311).

	Men (n = 10,276)		Women (n = 12,035)	
	n	(%)	n	(%)
Participation in sport group				
Participants	2,450	(23.8)	2,513	(20.9)
Almost every day	228	(2.2)	182	(1.5)
2 or 3 times a week	647	(6.3)	923	(7.7)
Once a week	497	(4.8)	811	(6.7)
Once or twice a month	474	(4.6)	347	(2.9)
A few times a year	604	(5.9)	250	(2.1)
Non-participants (Never)	6,084	(59.2)	6,762	(56.2)
Missing	1,742	(17.0)	2,760	(22.9)
Childhood SEP				
High (high or middle-high)	937	(9.1)	1,942	(16.1)
Middle	3,740	(36.4)	5,155	(42.8)
Low (middle-low or low)	5,057	(49.2)	4,173	(34.7)
Missing	542	(5.3)	765	(6.4)
Height*				
Tall	1,460	(14.2)	553	(4.6)
Middle-tall	2,687	(26.2)	2,051	(17.0)
Middle	3,292	(32.0)	3,986	(33.1)
Middle-short	1,745	(17.0)	3,394	(28.2)
Short	846	(8.2)	1,517	(12.6)
Missing	246	(2.4)	534	(4.4)
Educational level (years)				
≥13	2,250	(21.9)	1,472	(12.2)
10–12	3,365	(32.8)	4,324	(35.9)
<10	4,481	(43.6)	5,883	(48.9)
Missing	180	(1.8)	356	(3.0)
Annual equivalized income (yen)				
≥4.00 million	1,064	(10.4)	1,005	(8.4)
2.00–3.99 million	3,630	(35.3)	3,401	(28.3)
<2.00 million	4,272	(41.6)	4,820	(40.0)
Missing	1,310	(12.8)	2,809	(23.3)
Longest-held occupation				
Non-manual	3,367	(32.8)	1,099	(9.1)
Manual	5,905	(57.5)	7,473	(62.1)
None	54	(0.5)	1,061	(8.8)
Missing	950	(9.2)	2,402	(20.0)

SEP: socioeconomic position.

*Height in cm (men, women): tall (≥170, ≥160), middle-tall (165–169.9, 155–159.9), middle (160–164.9, 150–154.9), middle-short (155–159.9, 145–149.9), short (<155, <145).

**Table 2:** Adjusted prevalence ratio with 95% CI for association of childhood SEP with participation in sports groups in older Japanese men in the Japan Gerontological Evaluation Study (JAGES) 2010 survey with all variable multiple imputations (n = 10,276).

	Model 1		Model 2		Model 3		Model 4	
	PR (95% CI)	p	PR (95% CI)	p	PR (95% CI)	p	PR (95% CI)	p
*Childhood SEP*								
High	Reference		Reference		Reference		Reference	
Middle	0.93 (0.84–1.02)	0.136	0.93 (0.84–1.03)	0.175	0.98 (0.89–1.09)	0.714	0.99 (0.89–1.09)	0.826
Low	0.82 (0.74–0.91)	<0.001	0.83 (0.75–0.92)	<0.001	0.92 (0.83–1.02)	0.120	0.93 (0.84–1.04)	0.191

*Height*								
Tall			Reference		Reference		Reference	
Middle-tall			1.00 (0.92–1.11)	0.849	1.03 (0.93–1.13)	0.596	1.04 (0.94–1.14)	0.469
Middle			0.93 (0.84–1.02)	0.127	0.96 (0.87–1.06)	0.390	0.98 (0.89–1.07)	0.620
Middle-short			0.90 (0.80–1.01)	0.076	0.95 (0.85–1.07)	0.428	0.98 (0.87–1.10)	0.689
Short			0.81 (0.69–0.95)	0.010	0.87 (0.75–1.02)	0.094	0.91 (0.77–1.06)	0.223

*Education (years)*								
≥13					Reference		Reference	
10–12					0.90 (0.83–0.97)	0.005	0.93 (0.86–1.01)	0.083
<10					0.70 (0.64–0.76)	<0.001	0.75 (0.69–0.82)	<0.001

*Annual equivalized income*								
≥4.00 million yen							Reference	
2.00–3.99 million yen							0.94 (0.86–1.04)	0.225
<2.00 million yen							0.86 (0.78–0.95)	0.003

*Longest-held occupation*								
Non-manual							Reference	
Manual							0.86 (0.80–0.92)	<0.001
None							0.69 (0.36–1.33)	0.267

SEP: socioeconomic position; PR: prevalence ratio; CI: confidence interval.

Model 1: Adjusted for health-related factors (age, medication, instrumental activities of daily living, self-rated health, depression, body mass index, smoking status, alcohol intake, marital status, and social support).

Model 2: Model 1 + height.

Model 3: Model 2 + education.

Model 4: Model 3 + adulthood SEP (annual equivalized income, longest-held occupation).

**Table 3:** Adjusted prevalence ratio with 95% CI for association of childhood SEP with participation in sports groups in older Japanese women in the Japan Gerontological Evaluation Study (JAGES) 2010 survey with all variable multiple imputations (n = 12,035).

	Model 1		Model 2		Model 3		Model 4	
	PR (95% CI)	p	PR (95% CI)	p	PR (95% CI)	p	PR (95% CI)	p
*Childhood SEP*								
High	Reference		Reference		Reference		Reference	
Middle	0.98 (0.90–1.07)	0.668	0.99 (0.91–1.08)	0.785	1.04 (0.95–1.13)	0.433	1.03 (0.95–1.12)	0.485
Low	0.88 (0.80–0.97)	0.011	0.90 (0.82–0.99)	0.025	0.98 (0.89–1.08)	0.724	0.98 (0.89–1.08)	0.674

*Height*								
Tall			Reference		Reference		Reference	
Middle-tall			1.11 (0.95–1.29)	0.205	1.12 (0.96–1.31)	0.150	1.12 (0.96–1.31)	0.154
Middle			1.01 (0.87–1.18)	0.837	1.04 (0.89–1.21)	0.608	1.04 (0.89–1.21)	0.624
Middle-short			0.99 (0.85–1.16)	0.906	1.03 (0.88–1.20)	0.717	1.03 (0.88–1.21)	0.693
Short			0.85 (0.71–1.03)	0.091	0.90 (0.75–1.08)	0.242	0.90 (0.75–1.09)	0.280

*Education (years)*								
≥13					Reference		Reference	
10–12					0.91 (0.84–0.99)	0.032	0.92 (0.84–1.00)	0.057
<10					0.74 (0.68–0.82)	<0.001	0.77 (0.69–0.85)	<0.001

*Annual equivalized income*								
≥4.00 million yen							Reference	
2.00–3.99 million yen							1.09 (0.97–1.22)	0.144
<2.00 million yen							0.96 (0.86–1.07)	0.495

*Longest-held occupation*								
Non-manual							Reference	
Manual							0.95 (0.86–1.05)	0.307
None							0.91 (0.79–1.04)	0.169

SEP: socioeconomic position; PR: prevalence ratio; CI: confidence interval.

Model 1: Adjusted for health-related factors (age, medication, instrumental activities of daily living, self-rated health, depression, body mass index, smoking status, alcohol intake, marital status, and social support).

Model 2: Model 1 + height.

Model 3: Model 2 + education.

Model 4: Model 3 + adulthood SEP (annual equivalized income, longest-held occupation).

### 2.6 Statistical analysis

Because sex has been shown to influence the relationship between childhood SEP and physical inactivity (Cheval et al., 2018)—and because, as noted by Hawkes et al. (2013), disaggregation by sex is essential in health research—sex was controlled by conducting stratified analysis.

To account for potential biases due to missing values, we conducted multiple imputation analyses with 22,311 study participants, who experienced no limitations in activities of daily living. Following Sterne et al. (2009), all variables included in the analysis, such as the outcome variables, explanatory variables, and covariates, were imputed. Table 1 and Supplementary Table 1 presents the number of participants for whom data was imputed (because of missing values). Under a missing-at-random assumption, we created 20 imputed data using a chained equation procedure (White et al., 2011). The estimated parameters were combined using Rubin’s combination method (Rubin, 1987). Poisson regression with robust variance was used to examine the association between childhood SEP and sports group participation due to the relatively high prevalence of the latter (>10%): in such cases, odds ratios obtained from logistic regression models can significantly overestimate prevalence ratios (Barros and Hirakata, 2003, McNutt et al., 2003).

Model 1 was first adjusted for health-related factors (age, current medical treatment, IADL, self-rated health, depression, BMI, smoking status, alcohol intake, marital status, providing or receiving emotional social support). Next, Model 2 added height as a childhood circumstance to investigate how much it changes the association. In addition, since several studies indicated that educational attainment resulted in attenuation of the associations between low childhood SEP and adverse health outcomes later in life (Frenz et al., 2017, Galobardes et al., 2008, Lawlor et al., 2006), educational attainment was further adjusted in Model 3 to examine whether it influences these associations. Additionally, adulthood SEP (annual equivalized income and longest-held occupations) was added to Model 3 to investigate the effect on participants of all SEP (Model 4).

For sensitivity analyses (as a complete case analysis), different multiple imputation analyses were performed for participants, which excluded the missing values for sports group participation (outcome) and childhood SEP (explanatory) variables (Supplementary Tables 2 and 3). In addition, sensitivity analyses with the cut-off setting of “once or twice a month” for sports group participation were performed (Supplementary Tables 4 and 5). All statistical analyses and multiple imputations were performed using Stata/SE version 15.1 (StataCorp LLC, College Station, TX, USA) with statistical significance inferred at a two-tailed p-value of <0.05.

## 3 Results

Among all participants including missing values, 22.2% were sports group participants (Table 1), and 49.2% of men and 34.7% of women reported low or middle-low SEP in childhood, while 9.1% of men and 16.1% of women reported high or middle-high childhood SEP. Among men, the percentages for 13 or more years’ educational attainment and non-manual occupation were higher than among women.

The sociodemographic and health characteristics of the participants are shown in Supplemental Table 1. The mean age (standard deviation) was 73.9 (6.1) years (ranging from 65 to 101 years); 46.1% were men. Among men, being married was higher than among women. By contrast, compared to men, more women were non-smokers, non-drinkers, and had good IADL.

Table 2 shows the association between childhood SEP and the prevalence ratio (PR) of sports group participation in older men. Compared with the high childhood SEP group, the PR of sports group participation was 7% lower in the middle childhood SEP group and 18% lower in the low childhood SEP group in the health-related factors-adjusted model (Model 1). When analyses were controlled for height, the point estimates of PR for participation in sports groups were very slightly attenuated (Model 2). However, when analyses were controlled for educational attainment, the PR in the childhood SEP group was greatly attenuated (PR = 0.92, Model 3), and statistical significance disappeared. This association was very slightly attenuated in the low childhood SEP group after adjusting for adulthood SEP (Model 4).

When analyses were controlled for height, the point estimates of PR for participation in sports groups were slightly attenuated, although statistical significance remained the same (Model 2).

Among women, compared with the high childhood SEP group, the PR of sports group participation was 12% lower in the low childhood SEP group in the health-related factors-adjusted model (Model 1 in Table 3). When analyses were controlled for height, the point estimates of PR for participation in sports groups were slightly attenuated, although statistical significance remained the same (Model 2). However, when analyses were controlled for educational attainment, this association was no longer statistically significant (PR = 0.98, Model 3 in Table 3). The association was almost unchanged after adjusting for adulthood SEP (Model 4 in Table 3).

The sensitivity analysis that excluded the missing values for sports group participation and childhood SEP variables exhibited similar results with slightly smaller PRs (Supplemental Table 2, Table 3). The sensitivity analyses with the cut-off setting of “once or twice a month” revealed that the point estimates of PR exhibited similar results with a cut-off setting of “participants or non-participants” among men and women (Supplemental Tables 4 and 5).

## 4 Discussion

This study investigated the association between childhood SEP and sports group participation in older adults. Its results demonstrate that low childhood SEP is associated with lower sports group participation in older men, even after adjusting for health-related factors. After adjustment for educational attainment, the PR in the low childhood SEP group was both greatly attenuated and more attenuated than when adjusted for any other health-related or social characteristic in adulthood. This suggests that education may possibly shrink differences in the association between childhood SEP and sports group participation among older adults.

Despite inconsistencies in the results, prior evidence suggests that low childhood SEP groups participate less frequently in leisure-time PA in adulthood and early old age compared with high childhood SEP groups (Elhakeem et al., 2015, Elhakeem et al., 2017). Consistent with these studies, our study of older adults found that lower childhood SEP groups were less likely to participate in sports groups. While both men and women showed similar results, PR was consistently lower for men than women. One possible explanation is a sex difference in the tracking of PA from childhood to adulthood. Several previous studies have reported that men show greater stability in tracking PA compared to women in all phases of the life course (Telama et al., 2014). This difference is supported by findings that many life events, such as pregnancy, getting married, or having small children, have a greater influence on the PA of women than on that of men (Allender et al., 2008, Engberg et al., 2012, Telama et al., 2014). In addition, it has been suggested that men are more likely to participate in PA than women in childhood (World Health Organization, 2018), and also that men from lower SEP groups are more likely than women to engage in risky health behaviors, such as smoking, an unhealthy diet, and physical inactivity (Lawlor et al., 2006), which may be further explanations for the sex difference.

Our findings demonstrate that educational attainment has a stronger effect than other factors in attenuating the association of low childhood SEP with lower sports group participation in older adults. Our findings showed that the PR for participation in sports groups remained almost unchanged after adjusting for height, a variable often utilized as a proxy for childhood nutrition. In contrast, after adjusting for educational attainment, the PR in the low childhood SEP group was greatly attenuated. This is consistent with many previous studies (Elhakeem et al., 2015, Gidlow et al., 2006). Education is completed early in the life course and associated with subsequent income, employment, social networks, and behaviors (Byhoff et al., 2017). Moreover, educational attainment is associated with numerous mental and physical health outcomes (Byhoff et al., 2017, Kubota et al., 2017, Ladin, 2008, Xu et al., 2016). Furthermore, those who achieve higher educational attainment might have pursued a healthy lifestyle regardless of their personal income changes (Montez and Friedman, 2015). Our findings support the importance of education in explaining the link between childhood SEP and PA in adulthood (Elhakeem et al., 2015), and show that education also explains the link between childhood SEP and sports group participation in older adults. In the model, after adjusting for adulthood SEP, the PR remained almost unchanged. This suggest that education more strongly mediates the association between childhood SEP and sports group participation in adulthood than adulthood SEP. Thus, although education mediates the association between childhood SEP later-life sports participation, further study is called for to examine the indirect effects mediating educational attainment.

The mechanisms that explain the association between childhood SEP and later-life sports participation are not fully understood. However, given the similarity of the association between childhood SEP and PA (Elhakeem et al., 2015), educational attainment that increases health literacy may be one possible pathway (Montez and Friedman, 2015). Lower childhood SEP tends to restrict future SEP (Byhoff et al., 2017), as mentioned above. Another pathway may be the tracking of PA. Numerous studies consistently show that children from families with low SEP participate less in sports groups compared to high- SEP children (Stalsberg & Pedersen, 2010), and participation in PA and sports in childhood tends to be maintained throughout adulthood (Cleland et al., 2012, Telama et al., 2014) and thus old age. For these reasons, intervention to enhance educational attainment and promote sports in childhood may be an effective investment to increase sports group participation in future older adults. These childhood investments may lead to extending healthy life expectancy in the future. Additional studies conducting a mediation analysis are needed in order to demonstrate these associations and clarify the mechanism driving childhood SEP and sports group participation later in life.

### 4.1 Strengths and lsimitations

This study’s strengths include the large sample, comprising older adults from across Japan, and the inclusion of a wide range of variables. To our knowledge, no prior study has investigated the association between childhood SEP and sports group participation in older adults. Our findings establish childhood SEP as a new factor associated with sports group participation among Japanese older adults.

However, several limitations of this study should be considered. First, childhood SEP was evaluated retrospectively due to the cross-sectional design and self-reported method and is therefore susceptible to recall bias and could not establish causality. However, previous studies have confirmed the validity of retrospectively evaluating subjective childhood SEP (Ward, 2011) and childhood neighborhood context (Osypuk et al., 2015). In addition, the childhood subjective SEP was found to correlate with other objective indicators, of deprivation, such as height and SEP achieved in adulthood (Sakurai et al., 2010). Further studies are needed to examine whether there is a similar association between objective childhood SEP and participation in sports groups in old age. Second, since all measures were self-reported and the health status of some items such as smoking status and alcohol intake did not have detailed information, it is possible that measurement error occurred. Third, for the sampling method, while randomization was used in urban areas, the questionnaire was given to all eligible residents in the rural areas due to the small number of residents. Fourth, the generalizability of the results requires attention since this study did not include older adults with long-term-care insurance. Finally, information on participation in PA and in sports groups, including intensity, frequency, and types, was lacking. It would be useful to understand the mechanisms whereby childhood SEP affects sports group participation in old age. Therefore, further robust studies, including participation in PA and sports group participation at the early life stage, are needed to clarify this association.

## 5 Conclusion

This study demonstrated that low childhood SEP is associated with lower participation in sports groups among older adults, although this association may be attenuated by education. Our study highlighted the importance of education and implementing policies to tackle child poverty in order to increase sports group participation across the life course.

## CRediT authorship contribution statement

**Mitsuya Yamakita:** Conceptualization, Methodology, Formal analysis, Writing - original draft. **Satoru Kanamori:** Methodology, Investigation, Writing - review & editing. **Naoki Kondo:** Data curation, Formal analysis, Writing - review & editing, Supervision, Funding acquisition, Project administration. **Toyo Ashida:** Methodology, Writing - review & editing. **Takeo Fujiwara:** Investigation, Methodology, Writing - review & editing, Supervision. **Taishi Tsuji:** Data curation, Methodology, Formal analysis. **Katsunori Kondo:** Conceptualization, Methodology, Investigation, Writing - review & editing, Supervision, Funding acquisition, Project administration.
