# Supplementary material for: Association between childhood socioeconomic position and sports group participation among Japanese older adults: A cross-sectional study from the JAGES 2010 survey
Source: Prev Med Rep. 2020 Feb 17;18:101065. doi: 10.1016/j.pmedr.2020.101065 (PMC7038007; doi:10.1016/j.pmedr.2020.101065)
Supplement: Supplementary data 1 [file mmc1.docx]

**Supplementary Table 1.** The sociodemographic and health characteristics of the participants

**Supplementary Table 2.** Adjusted prevalence ratio with 95% CI for association of childhood SEP with participation in sports groups in older Japanese men in the Japan Gerontological Evaluation Study (JAGES) 2010 survey with complete case analysis for participation in sports groups and childhood SEP (n=8,123).

CI: confidence interval; SEP: socioeconomic position; PR: prevalence ratio.

Model 1: Adjusted for health-related factors (age, medication, instrumental activities of daily living, self-rated health, depression, body mass index, smoking status, alcohol intake, marital status, and social support).

Model 2: Model 1 + height

Model 3: Model 2 + education

Model 4: Model 3 + adulthood SEP (annual equivalized income, longest-held occupation)

**Supplementary Table 3.** Adjusted prevalence ratio with 95% CI for association of childhood SEP with participation in sports groups in older Japanese women in the Japan Gerontological Evaluation Study (JAGES) 2010 survey with complete case analysis for participation in sports groups and childhood SEP (n=8,786).

CI: confidence interval; SEP: socioeconomic position; PR: prevalence ratio.

Model 1: Adjusted for health-related factors (age, medication, instrumental activities of daily living, self-rated health, depression, body mass index, smoking status, alcohol intake, marital status, and social support).

Model 2: Model 1 + height

Model 3: Model 2 + education

Model 4: Model 3 + adulthood SEP (annual equivalized income, longest-held occupation)

**Supplementary Table 4**. Adjusted prevalence ratio with 95% CI for association of childhood SEP with participation (less than once or twice a month) in sports groups in older Japanese men in the Japan Gerontological Evaluation Study (JAGES) 2010 survey with multiple imputations (n=10,276).

CI: confidence interval; SEP: socioeconomic position; PR: prevalence ratio.

Model 1: Adjusted for health-related factors (age, medication, instrumental activities of daily living, self-rated health, depression, body mass index, smoking status, alcohol intake, marital status, and social support).

Model 2: Model 1 + height

Model 3: Model 2 + education

Model 4: Model 3 + adulthood SEP (annual equivalized income, longest-held occupation)

**Supplementary Table 5** Adjusted prevalence ratio with 95% CI for association of childhood SEP with participation (less than once or twice a month) in sports groups in older Japanese women in the Japan Gerontological Evaluation Study (JAGES) 2010 survey with multiple imputations (n=12,035).

CI: confidence interval; SEP: socioeconomic position; PR: prevalence ratio.

Model 1: Adjusted for health-related factors (age, medication, instrumental activities of daily living, self-rated health, depression, body mass index, smoking status, alcohol intake, marital status, and social support).

Model 2: Model 1 + height

Model 3: Model 2 + education

Model 4: Model 3 + adulthood SEP (annual equivalized income, longest-held occupation)
